# Optofluid-Based Reflective Displays

**DOI:** 10.3390/mi9040159

**Published:** 2018-04-01

**Authors:** Mingliang Jin, Shitao Shen, Zichuan Yi, Guofu Zhou, Lingling Shui

**Affiliations:** 1 National Center for International Research on Green Optoelectronics, South China Normal University, Guangzhou 510006, China; jinml@scnu.edu.cn (M.J.); shenshitao@m.scnu.edu.cn (S.S.); guofu.zhou@m.scnu.edu.cn (G.Z.); 2 Guangdong Provincial Key Laboratory of Optical Information Materials and Technology and Institute of Electronic Paper Displays, South China Academy of Advanced Optoelectronics, South China Normal University, Guangzhou 510006, China; 3 Zhongshan Institute, University of Electronic Science and Technology of China, Zhongshan 528402, China; yizichuan@zsc.edu.cn

**Keywords:** reflective display, electrowetting, electrophoresis, interferometric modulator, MEMS

## Abstract

Displays can present information like text, images, or videos in a different color (visible light) by activating the materials in pixels. In a display device, pixels are typically of micrometer size and filled with displaying materials that are aligned and controlled by a display driver integrated circuit. Typical reflective displays can show designed information by manipulating ambient light via the microfluidic behavior in pixels driven by electrophoresis, electrowetting, or electromechanical forces. In this review, we describe the basic working principles and device structures of three reflective displays of electrophoresis display (EPD), electrowetting display (EWD), and interferometric modulator display (IMOD). The optofluidic behavior and controlling factors relating to the display performance are summarized.

## 1. Introduction

Displays show information on demand by manipulating visible light via changing the colors of one material or moving different colored materials at the microscale pixels. Depending on the interaction between light and materials, visible light can be manifested by different materials via reflection, transmission, or emission. Liquid crystal display (LCD), organic light emission display (OLED), and electrophoretic display (EPD) are the representatives of transmissive display, emissive display, and reflective display, respectively. In reflective displays, visible light with a wavelength in the range of about 400–700 nm could be either reflected, scattered, or absorbed by the displaying materials in pixels. Each pixel with a size in the range of nano- to micrometers is controlled by a corresponding electrical backplane, showing either different colors or grayscale (degree of black-and-white) [1,2,3].

Reflective display is also named “electronic paper,” which possesses both the speed of “electronics” and the reading comfort of “paper.” Thus, it has been given a lot of attention over the last half-century. The commercially available Kindle e-Paper and electronic batch are successful examples of EPD [4,5,6].

**Figure 1:**
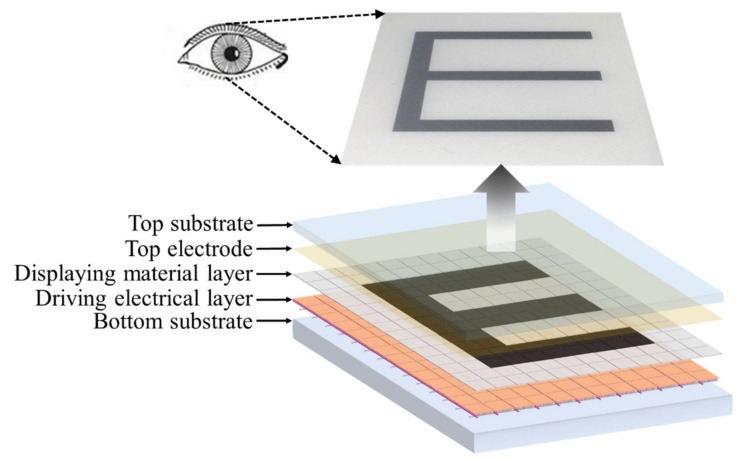
Schematic of a reflective display device.

A schematic drawing of a reflective display device is shown in Figure 1. It consists of basic components (from bottom to top) of bottom substrate → driving electrical layer (thin film transistors or electrodes) → displaying material layer (in pixels or not) → top electrode(s) → top (cover) substrate including protective and optical films.

Pixel size in display device is typically in the micrometer range at the horizontal *x*- and *y*-axis, and nanometer to micrometer in the vertical *z*-axis. Thus, human eyes cannot directly distinguish pixels from each other. However, once an area of pixels is actuated to demonstrate the same color, obvious light/color differences are visible by eyes. Each pixel can be driven by a display backplane individually to achieve a complex information display. For instance, a black “*E*” could be shown on a white background by only driving the pixels in the “*E*” area to black color. From this point of view, each pixel can be regarded as an optical switch. With reversible control of the displaying materials, the numbers and positions of the pixels will determine the content of displayed information. Therefore, the switching (open and closed) speed and degree determine the quality of a display device in speed and contrast ratio, respectively.

In this work, we define “optofluid-based reflective display” as a reflective display using interactions between light (visible light) and fluids (e.g., dispersion in microcapsule for EPD, dual-fluid in micropixel for EWD, and air in microcavity for IMOD). The electrochemical reaction (for electrochromic display) and molecular re-arrangement (for liquid crystal display) driven by the electric field are excluded. In an electrophoretic display (EPD), black and white particles suspended in an insulating liquid medium are driven by electrophoresis to move up and down to show black, white, or gray [7]. In an electrowetting display (EWD), dye dissolved organic or aqueous solutions are driven to move by electrowetting to display the color of the liquid solution or the background substrate [2,8]. In an interferometric modulator display (IMOD), the incident light is reflected by the metal bottom surface and the stacked film, showing the color of the constructively interfered wavelength by controlling the height of the air cavity via electromechanical force [3].

## 2. Electrophoretic Display

Electrophoretic display was first presented in 1973 [5] by placing two bi-chromo-color pigments in glass cavities and controlling the movement of particles to switch between two colors. Electrophoretic displays have been the subject of intense research for many years because of their wide marketing potential [7,9,10]. Currently, EPDs have been widely used in the fields of electronic books, electronic labels, and smart watches [4,11].

**Figure 2:**
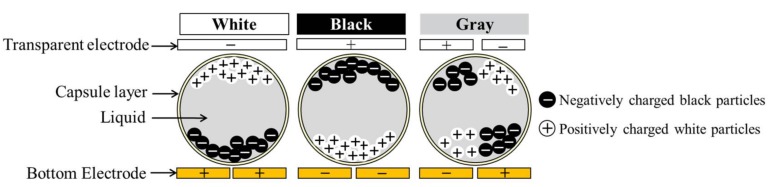
Working principle of the microcapsule-based electrophoretic display.

The major breakthrough of EPDs was made in 1998 by Jacobson et al. [7], who employed electrophoretic materials and device design to overcome the critical shortcomings of EPDs at that time. The mature and most successful EPD mode is named microcapsule-based EPD, in which the dispersion of black and white nanoparticles is encapsulated in microscale capsules and driven by pixelated electric backplane. The microcapsule-based EPD is shown in Figure 2. The black and white particles encapsulated in the microcapsules are positively and negatively charged, respectively. The microcapsules are sandwiched between the top transparent electrode and bottom pixel electrodes. The pixel electrodes can provide alternative voltages to actuate white and black particles to move in the microcapsules along the direction of electric field. In order to achieve accurate gray tones, the applied voltage on a common electrode can be adjusted. Therefore, display performance like grayscale or contrast ration is closely related to the applied voltage.

In an EPD pixel (microcapsule), typically, five chemical materials exist: pixel wall (or microcapsule shell), insulating liquid, colored pigments (particles), charge control agent, and stabilizer. Electrophoresis causes the movement of charged particles through the stationary insulating liquid. The conventional Helmholtz–Smoluchowski equation is applied in EPDs [12]:

(1) $$U=\;\frac{\varepsilon \xi_{EP}E_{x}}{\mu }$$

where *U* is the electrophoretic velocity of the particle, *ε* is the dielectric constant of the insulating liquid, *ξ_EP_* is the zeta potential of the particle, *E_x_* is the applied electrical field, and *μ* is the mobility of the particle. The electrophoretic zeta potential (*ξ_EP_*) is a property of the charged particle. Except for electrophoretically driven particle moving, a stabilizer is used to keep positively charged white particles and negatively charged black particles separate from each. Therefore, the electrophoretic particles are typically core-shell structure with white or black cores encapsulated by polymeric shells. Moreover, by tuning the coating polymeric structure, the density difference between the insulating liquid, black particles, and white particles could be further manipulated to form a stable product for long-term usage.

The displaying material in EPDs is a colloidal suspension that is similar to painting and printing inks; it thus allows for comfortable readability in natural environmental conditions with a similar contrast ratio to newspapers and a reflectivity of ~40%. For the same reason, the displaying materials of EPDs are called “E-ink.” Moreover, the microscale encapsulation minimizes the gravity effect; thus, the force balance without external voltage could be achieved. Therefore, EPDs possess the advantage of “bi-stability” for low energy consumption. Generally, an e-book reader can work for two weeks without recharging, and an image can be maintained for years without driving actuation. Furthermore, the spherical shape of the microcapsules makes the view angle wide without an obvious difference. However, the display speed of ~10 fps, with just several options of ink colors, has limited its applications. On the other hand, flexible displays could be easily fabricated using E-ink materials through wet-printing technologies. This makes it possible for wider applications in wearable electronic devices and electronic skins.

## 3. Electrowetting Display

The electrowetting principle, as applied in display devices, was first demonstrated in 1981 by Beni et al. [13,14,15,16]. The working principle of EWD is based on electrowetting driving microdroplets to move laterally or vertically. A practical and well-functional electrowetting display device based on colored oil driven by conductive liquid was successfully demonstrated in 2003 by Hayes and Feenstra at Philips Research Lab [2]. EWDs attracted much attention thereafter. In 2009, a brilliant EWD was presented by Heikenfeld et al., in which pigment dispersions were driven to move vertically between two layers in a multilayer structure [8].

**Figure 3:**
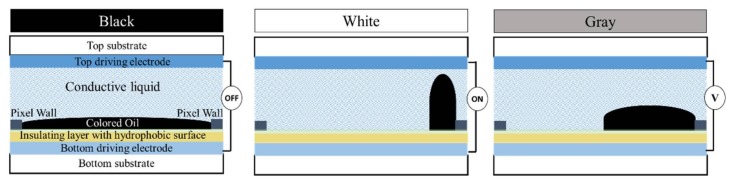
Device structure and working principle of the electrowetting display.

A schematic structure of the dual microfluidic EWD is shown in Figure 3. It consists of a bottom substrate covered with pixel electrodes, hydrophobic insulating layer, hydrophilic pixel walls, colored oil (displaying material), conductive liquid, and top electrodes attached on the top substrate. At the stage of 0 voltage, the colored oil forms a continuous film in a pixel between the hydrophobic insulator (connected with the bottom electrode) and the conductive liquid (connected with the top electrode) due to the hydrophobic interaction between the oil and hydrophobic coating. At this stage, the pixel shows the color of the oil. When voltage V is applied between the top and bottom electrodes, the energetically stable state is destroyed since an electrostatic force is added. Water (a conductive liquid) moves towards the bottom surface driven by electrowetting, breaking and pushing the oil film aside to pixel walls and corners. At this stage, the pixel shows the color of the bottom substrate when viewed from the top. In this way, the optical properties of the stack are tuned between an OFF-state color (e.g., black oil) and an ON-state color (e.g., white substrate). In general, specific grayscale can be achieved by the oil film coverage ratio over a pixel, being controlled by the applied voltage across the top and bottom electrodes. Overall, a simple and highly reversible optical switch in a pixel is obtained, driven by electrowetting. These microscale pixels are arranged as demonstrated in Figure 1 to achieve the information display.

The performance of a EWD is highly dependent on the device’s geometry and the properties of the properties of its constructive materials of the device, including an insulating layer, a hydrophobic layer, oil, and a conductive liquid. Based on the classical theory of electrowetting, the reduction of the contact angle is induced by the electrostatic force when a voltage is applied between the conductive fluid and electrode underneath [17]. When the applied voltage changes from 0 to *V*, an obvious force balance is rebuilt to induce the contact angle *θ* to change from *θ*_0_ to *θ_V_*. The electrostatic force is balanced by the surface energy. The electrostatic force is calculated by *CV*^2^/2, where *C* is the capacitance between the electrode and the conductive liquid. As the electrode is covered by a hydrophobic insulating layer with a thickness of *d*, the capacitance is calculated as *C* = *ε*_0_*ε_r_*/*d* (*ε*_0_ and *ε_r_* are the dielectric constants of air and the insulating material, respectively). Therefore, the obtained contact angle at voltage *V* (*θ_V_*) is calculated by the Young–Lippmann equation [2,17]:

(2) $$cos\theta_{V}=cos\theta_{0}+\frac{\varepsilon_{0}.\varepsilon_{r}}{2\gamma_{wo}d}V^{2},$$

where *r_wo_* is the interfacial tension between conductive water phase and insulating oil phase. This equation has been successfully employed by many investigators in correlating experimental results with theory for a significant change in contact angles.

With the effort of scientists from both research institutions and companies, EWD has shown its potential for high-quality information displays. It has presented several advantages: (1) quick response with a switching speed <20 ms (>50 fps) for video display [18]; (2) good optical performance with >50% white state reflectance and full color range [2,8], and (3) the possibility of flexible displays due to the fully flexible fluidic display materials.

## 4. Interferometric Modulator Display

**Figure 4:**
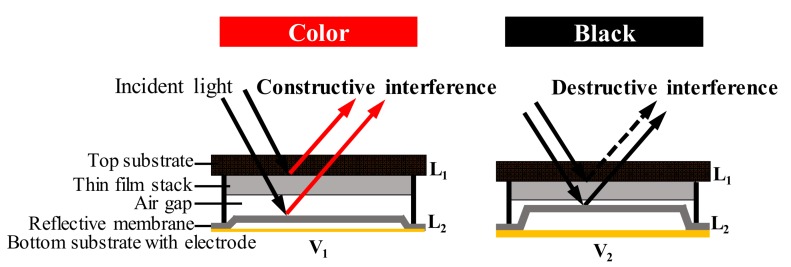
Device structure and working principle of the interferometric modulator display (IMOD).

The interferometric modulator display (IMOD) is based on micro-electromechanical systems (MEMS) technology developed by the Iridigm Display Corporation (later merged into Qualcomm), which is also known as the Mirasol display [19,20]. The key of an IMOD is the tunable optical cavity. As shown in Figure 4, each optical cavity is composed of a self-supporting deformable reflective membrane on the bottom electrode and a thin-film stack residing on a transparent top substrate. Each stacks acts as one mirror of an optically resonant cavity. The deformable membrane is used to adjust the geometry of the cavity, which serves as the displaying pixel. When ambient light hits the structure, it is reflected off both the top of the thin-film stack (*L*_1_) and the reflective membrane (*L*_2_). Depending on the height of the cavity, reflected light of a certain wavelength will be slightly out of phase when reflecting off the membrane and the thin film. Therefore, some wavelengths will either constructively or destructively interfere depending on the phase difference. Human eyes will receive one color of a certain wavelength (e.g., red), which will be amplified by constructive interference with respect to others. However, the destructive interference results in a dark state (black). When a voltage is applied, the flexible membrane will be attracted up to tune the gap height driven by the electromechanical force. In this way, the displayed color (light) is selectively manipulated by the applied driving voltage.

An IMOD pixel is an optically resonant cavity similar to a Fabry–Pérot interferometer (etalon). When ambient light enters this cavity and reflects off the thin-film mirror, it interferes with itself, generating a resonant color determined by the height of the cavity. Therefore, the requirement for a Fabry–Pérot etalon is applied for the IMOD, for which,

(3) $$h=m\left(\frac{\lambda }{2}\right),$$

where *h* is the height of the cavity, *m* is an integer, and *λ* is the wavelength of the light inside the cavity. In this way, the light reflection is controlled by the fluidic medium (the air cavity) to achieve the optofluidic display. A practical IMOD is composed of thousands of pixels with primary colors of red (R), green (G), and blue (B). A single RGB pixel is built from one or more subpixels. A monochromatic array of subpixels represents different levels for each color, and for pixels [19,20]. Therefore, in IMOD, by tuning the fluidic airgap through the applied voltage, not only is the displayed color selected from the ambient light (white), but also the brightness is determined depending on the uniformity of the constructed devices [3].

Depending on the electric field applied between the substrate and the thin film, the film can be positioned in one state that can remain until the next refresh [3,21]. Thus, a bi-stable reflective display is achieved. Flexible displays are also feasible with this technology by employing a flexible polyethylene naphthalate (PEN) film as the bottom substrate and suspended upper substrate [22]. However, due to the low production yield caused by sophisticated fabrication and a low display speed (~40 fps), IMOD was outside the scope of mainstream technology after a short period of “flash.”

## 5. Conclusions and Outlook

Controlling the optical performance of displays via microfluidic behavior is one of the key areas of optofluidics. In this review, we selectively review three popular reflective display mechanisms using micro-confined fluidic mediums as displaying materials, including electrophoretic display (EPD), electrowetting display (EWD), and interferometric modulator display (IMOD). The driving forces are electrophoretic, electrowetting, and electro-mechanical forces in EPD, EWD, and IMOD, respectively. The microfluid-based displaying mediums are the nanoparticle dispersion, oil-water bifluidic system, and air cavity in EPD, EWD, and IMOD. The optical performance of these three display technologies is determined by the fluidic behavior confined in the pixels of each display device.

Nowadays, displays are everywhere in our life as key components of mobile phones, computers, televisions, etc. Reflective displays make use of ambient light, thus having advantages of comfortable readability in bright and outdoor environments, and energy savings according to bi-stable display and no backlight. In the future, with better control of fluidic properties (viscosity, surface tension, refractive index, conductivity, and dielectric constant) and the development of device design and fabrication, these optofluid-based reflective displays could achieve faster responses and higher optical contrast. New optofluidic devices and high-quality flexible displays can also be expected according to the fully flexible display materials based on fluids.
